# The protective association of endogenous immunoglobulins against sepsis mortality is restricted to patients with moderate organ failure

**DOI:** 10.1186/s13613-017-0268-3

**Published:** 2017-04-20

**Authors:** Ignacio Martin-Loeches, Arturo Muriel-Bombín, Ricard Ferrer, Antonio Artigas, Jordi Sole-Violan, Leonardo Lorente, David Andaluz-Ojeda, Adriele Prina-Mello, Ruben Herrán-Monge, Borja Suberviola, Ana Rodriguez-Fernandez, Pedro Merino, Ana M. Loza, Pablo Garcia-Olivares, Eduardo Anton, Eduardo Tamayo, Wysali Trapiello, Jesús Blanco, Jesús F. Bermejo-Martin, the GRECIA group, Marta María García-García, Mª Jesús López Pueyo, Jose Antonio Fernandez Ratero, Miguel Martinez Barrios, Fernando Callejo Torre, Sergio Ossa Echeverri, Demetrio Carriedo Ule, Ana Mª Domínguez Berrot, Fco Javier Díaz Domínguez, Susana Moradillo, Braulio Alvarez Martínez, Noelia Albalá, Juan Carlos Ballesteros, Marta Paz Perez, Elena Perez Losada, Santiago Macías, Rafael Pajares García, Noelia Recio García Cervigón, Mª Mar Gobernado Serrano, Mª José Fernández Calavia, Daniel Moreno Torres, Concha Tarancón, Teresa Loreto, Priscila Carcelen, Rafael Cítores y Francisco Gandía

**Affiliations:** 1 Multidisciplinary Intensive Care Research Organization (MICRO), St. James’s University Hospitals Dublin, James’s St, Ushers, Dublin 8, Ireland; 2 Intensive Care Department, Hospital Universitario Río Hortega de Valladolid, Calle Dulzaina, 2, 47012 Valladolid, Spain; 3 Intensive Care Department, Vall d’Hebron University Hospital, Shock Organ Dysfunction and Resuscitation Research Group, Vall d’ Hebron Research Institute, Passeig de la Vall d’Hebron, 119-129, 08035 Barcelona, Spain; 4 ABISS-Edusepsis (AntiBiotic Intervention in Severe Sepsis), Intensive Care Department, Vall d’Hebron University Hospital, Passeig de la Vall d’Hebron, 119-129, 08035 Barcelona, Spain; 5 Critical Care Center, Corporacion Sanitaria Universitaria Parc Tauli, Autonomous University of Barcelona, Parc Taulí 1, 08208 Sabadell, Barcelona, Spain; 6 CIBER Enfermedades Respiratorias, Instituto de Salud Carlos III, C/Sinesio Delgado,4, 28029 Madrid, Spain; 7 Critical Care Medicine Service, Hospital Dr Negrin, Barranco de la Ballena, s/n, 35010 Las Palmas de Gran Canaria, Las Palmas, Spain; 8 Critical Care Medicine Service, Hospital Universitario de Canarias, Carretera de Ofra, s/n, 38320 San Cristóbal de La Laguna, Santa Cruz De Tenerife, Spain; 9 Critical Care Medicine Service, Hospital Clínico Universitario de Valladolid, Av. Ramón y Cajal, 3, 47003 Valladolid, Spain; 10 Laboratory for Biological Characterisation of Advanced Materials, Trinity Translational Medicine Institute (TTMI), James’s St, Dublin 8, Ireland; 11 Critical Care Medicine Service, Hospital Universitario Marqués de Valdecilla, Av. Valdecilla, 25, 39008 Santander, Spain; 12 Microbiology Service, Hospital Marques de Valdecilla, Av. Valdecilla, 25, 39008 Santander, Spain; 13 Critical Care Medicine Service, Hospital Nuestra Señora de Valme, Av. de Bellavista, s/n, 41014 Seville, Spain; 14 Critical Care Medicine Service, Hospital General Universitario Gregorio Marañón, Calle del Dr. Esquerdo, 46, 28007 Madrid, Spain; 15 Critical Care Medicine Service, Hospital de Manacor, Carretera Manacor Alcudia, s/n, 07500 Manacor, Spain; 16 Anesthesiology Service, Hospital Clínico Universitario de Valladolid, Av. Ramón y Cajal, 47003 Valladolid, Spain; 17 BIO∙SEPSIS (Laboratory of Biomedical Research in Sepsis), Hospital Clínico Universitario, SACYL, Av. Ramón y Cajal 3, 47003 Valladolid, Spain; 18 GRECIA Group (Grupo de Estudios y Análisis en Cuidados Intensivos), Critical Care Medicine Service, Hospital Universitario Río Hortega de Valladolid, Calle Dulzaina, 2, 47012 Valladolid, Spain

**Keywords:** Immunoglobulins, Sepsis, Severity, Survival

## Abstract

**Background:**

Pre-evaluation of endogenous immunoglobulin levels is a potential strategy to improve the results of intravenous immunoglobulins in sepsis, but more work has to be done to identify those patients who could benefit the most from this treatment. The objective of this study was to evaluate the impact of endogenous immunoglobulins on the mortality risk in sepsis depending on disease severity.

**Methods:**

This was a retrospective observational study including 278 patients admitted to the ICU with sepsis fulfilling the SEPSIS-3 criteria, coming from the Spanish GRECIA and ABISS-EDUSEPSIS cohorts. Patients were distributed into two groups depending on their Sequential Organ Failure Assessment score at ICU admission (SOFA < 8, *n* = 122 and SOFA ≥ 8, *n* = 156), and the association between immunoglobulin levels at ICU admission with mortality was studied in each group by Kaplan–Meier and multivariate logistic regression analysis.

**Results:**

ICU/hospital mortality in the SOFA < 8 group was 14.8/23.0%, compared to 30.1/35.3% in the SOFA ≥ 8 group. In the group with SOFA < 8, the simultaneous presence of total IgG < 407 mg/dl, IgM < 43 mg/dl and IgA < 219 mg/dl was associated with a reduction in the survival mean time of 6.6 days in the first 28 days and was a robust predictor of mortality risk either during the acute or during the post-acute phase of the disease (OR for ICU mortality: 13.79; OR for hospital mortality: 7.98). This predictive ability remained in the absence of prior immunosuppression (OR for ICU mortality: 17.53; OR for hospital mortality: 5.63). Total IgG < 407 mg/dl or IgG1 < 332 mg/dl was also an independent predictor of ICU mortality in this group. In contrast, in the SOFA ≥ 8 group, we found no immunoglobulin thresholds associated with neither ICU nor hospital mortality.

**Conclusions:**

Endogenous immunoglobulin levels may have a different impact on the mortality risk of sepsis patients based on their severity. In patients with moderate organ failure, the simultaneous presence of low levels of IgG, IgA and IgM was a consistent predictor of both acute and post-acute mortalities.

**Electronic supplementary material:**

The online version of this article (doi:10.1186/s13613-017-0268-3) contains supplementary material, which is available to authorized users.

## Background

Pre-evaluation of endogenous immunoglobulin levels has been proposed as a potential tool to identify sepsis patients deserving replacement treatment with intravenous immunoglobulins (IVIG), to improve its results in this severe condition [1]. Nonetheless, the impact of endogenous immunoglobulins levels on the risk of mortality in sepsis remains a controversial issue. A recent meta-analysis leaded by Shankar-Hari M found that the prevalence of IgG hypogammaglobulinemia on the day of sepsis diagnosis is as high as 70%, but this finding did not identify a subgroup of patients with a higher risk of death [2]. Recently, results from the SBITs (Score-based immunoglobulin G therapy of patients with sepsis) study showed that initial low IgG levels did not discriminate between survival and non-survival in patients with severe sepsis and septic shock [3]. In addition, patients with the highest IgG levels (fourth quartile) showed a statistically significant higher mortality in a risk-adjusted calculation compared to the reference quartile [3]. A previous report from our group supported that the answer could be in considering immunoglobulin isotypes not as isolated entities but in evaluating their prognostic ability in combination [4, 5].

There are a number of factors that in our opinion have not been appropriately addressed in the studies evaluating the predictive ability of immunoglobulins: (1) we have demonstrated in a recent article that disease severity strongly influences biomarker performance in sepsis [6]; (2) the influence of previous immunosuppression has not been evaluated [5]; (3) the impact of immunoglobulins on hospital mortality has not been sufficiently studied, with the majority of works being focused on the acute period of the disease [2]; (4) finally, the ability of endogenous immunoglobulin levels to predict mortality in patients fulfilling the SEPSIS-3 criteria [7, 8] has not been reported to the present moment.

Aimed by the need of identifying patient subsets that could benefit the most from IVIG therapy, we have now evaluated the ability of endogenous immunoglobulins levels and also of a combined immunoglobulin score to predict mortality risk of sepsis patients fulfilling the SEPSIS-3 diagnostic criteria in two different scenarios of disease severity, at the short and the long term, with and without presence of previous immunosuppression.

## Methods

### Study design

Patients from two multicenter epidemiological studies on sepsis were merged to evaluate, in a retrospective manner, the association between levels of endogenous immunoglobulins in plasma and mortality depending on disease severity at ICU admission. One hundred and eighty patients came from the GRECIA study [9] (Grupo de Estudios y Análisis en Cuidados Intensivos), and 98 came from the ABISS-Edusepsis study (AntiBiotic Intervention in Severe Sepsis) [10]. In both studies, patients had sepsis at the time of admission to the ICU. For this study, only those patients fulfilling the new definition proposed by the SEPSIS-3 Consensus were considered [7]. Patients with human immunodeficiency virus (HIV) infection and those undergoing radiotherapy or receiving immunosuppressive drugs, including chemotherapy or systemic steroids, in the last 3 months prior to admission to the ICU were considered to be immunosuppressed. Exclusion criteria were cardiac arrest, therapeutic effort limitation and lack of informed consent. A common data sheet was developed to collect the clinical data, including medical history, physical examination and hematological, biochemical, radiological and microbiological investigations from the two studies. Treatment decisions were not standardized for all patients but were made by the treating physician, always based upon the Surviving Sepsis Campaign guidelines recommendations.

### Immunoglobulin quantification

A 5-ml sample of blood was collected in an EDTA tube from all patients in the first 12 h following ICU admission. The blood was centrifuged, and plasma was obtained and stored at −80 °C until required for immunoglobulin quantification. Plasma levels of immunoglobulins were measured using a multiplex immunoglobulin isotyping kit (Biorad TM, Hercules, CA, USA) on a Luminex platform. All plasma samples from the two studies (GRECIA and ABISS) were tested for immunoglobulin concentrations using the same equipment, to avoid potential bias due to multiplatform testing.

### Statistical analysis

For the demographic and clinical characteristics of the patients, differences between groups were assessed using the Chi-square test for categorical variables and the Mann–Whitney *U* test for continuous variables when appropriate. Patients were split into two groups based upon the percentile 50 for the Sequential Organ Failure Assessment (SOFA) score at ICU admission. In consequence, two groups of patients were generated (SOFA < 8 and SOFA ≥ 8). Deciles of immunoglobulin concentrations were used to categorize patients below or above each decile, creating the corresponding categorical variables. Deciles were calculated for the entire cohort, since no differences for immunoglobulin levels were found between patients with SOFA score <8 and those with SOFA scores ≥8 (Table 1). We determined the occurrence of death in each severity group using Kaplan–Meier curves. Time was censored at day 28 following admission to the ICU for this analysis. The first decile showing significant differences between groups based on the log-rank test was considered as the immunoglobulin threshold. We established immunoscores (ISC) for identifying those patients with the combined presence of low levels of two or more immunoglobulins (below each respective threshold). An overall score of 0 was assigned to all patients with levels below the thresholds for all immunoglobulins forming each immunoscore and a score of 1 to the remaining patients. The dichotomous variables created for each immunoglobulin using the identified thresholds as well as the immunoscores were further introduced into a multivariate logistic regression analysis to determine the association between immunoglobulin levels and the risk of mortality at the ICU and also at the hospital. Those variables of Table 1 yielding *p* values <0.1 in the univariate analysis were considered as potential confounding factors and were further introduced in the multivariate one as adjusting variables. Data analysis was performed using SPSS for WINDOWS version 22.0 software (IBM-SPSS, Chicago, IL, USA).

**Table 1:** Clinical characteristics of the patients

	Total “*N*”: 278 patients	SOFA < 8 *N* = 122	SOFA ≥ 8 *N* = 156	*p* value
Characteristics	Cohort GRECIA/ABISS	81/41	99/57	n.s
	Age (years)	70.5 (18.0)	67.50 (19.0)	n.s
	Sex (male)	70 (57.4%)	112 (71.8%)	0.012
Comorbidities	Chronic cardiovascular disease	15 (12.3%)	21 (13.5%)	n.s
	Chronic respiratory disease	21 (17.2%)	29 (18.6%)	n.s
	Chronic renal failure	12 (9.80%)	16 (10.3%)	n.s
	Chronic hepatic failure	2 (1.6%)	11 (7.1%)	0.034
	Diabetes mellitus	23 (18.9%)	24 (15.4%)	n.s
	Immunosuppression	23 (18.9%)	39 (25.0%)	n.s
Severity and outcome	APACHE-II	18.0 (7.0)	23.0 (10.0)	0.001
	Sepsis with cardiovascular dysfunction	77 (63.1%)	147 (94.2%)	0.001
	Mortality at the ICU	18 (14.8%)	47 (30.1%)	0.003
	Mortality at the hospital	28 (23.0%)	55 (35.3%)	0.026
Source of infection	Respiratory	47 (40.2%)	59 (38.8%)	n.s
	Abdominal	38 (32.5%)	44 (29.1%)	n.s
	Urinary tract	14 (12.0%)	21 (13.9%)	n.s
	Bacteremia–catheter	3 (2.6%)	11 (7.3%)	n.s
	Other	15 (12.8%)	15 (9.9%)	n.s
	Microbiologically confirmed infection	57 (46.7%)	92 (59.0%)	0.047
Microbiology	Gram-negative bacteria	28 (23%)	55 (35.3%)	0.029
	Gram-positive bacteria	31 (25.4%)	35 (22.4%)	n.s
	Fungemia	6 (4.9%)	3 (1.9%)	n.s
	Other	4 (3.3%)	4 (2.6%)	n.s
	Polimicrobian sepsis	16 (13.1%)	11 (7.1%)	n.s
Laboratory parameters at diagnosis	White blood cells (cells/mm3)	15.800 (9500)	15.400 (15,080)	n.s
	Platelets/µl	210.000 (136,000)	133.000 (142,500)	0.001
	Potassium (mEq/l)	4.2 (0.8)	4.3 (1.0)	n.s
	Sodium (mEq/l)	138.0 (8.0)	139.0 (9.0)	n.s
	Glycemia (mg/dl)	167.0 (94.0)	170.0 (87.0)	n.s
	Creatinine (mg/dl)	1.2 (1.1)	2.0 (1.7)	0.001
	Albumin (g/dl)	2.3 (1.0)	2.3 (0.8)	n.s
	IgG total (mg/dl)	618.2 (516.0)	528.7 (469.0)	n.s
	IgG1 (mg/dl)	482.6 (370.5)	446.71 (377.9)	n.s
	IgG2 (mg/dl)	17.8 (28.2)	15.0 (21.2)	n.s
	IgG3 (mg/dl)	51.1 (76.0)	49.8 (62.0)	n.s
	IgG4 (mg/dl)	17.2 (46.5)	16.3 (35.0)	n.s
	IgM (mg/dl)	38.0 (37.4)	36.40 (32.1)	n.s
	IgA (mg/dl)	278.7 (343.0)	273.3 (375.6)	n.s

Continuous variables are shown as median (inter-quartile rank) and categorical variables as *n* (%)

*n.s.* not significant

## Results

### Clinical characteristics of the patients depending on disease severity at ICU admission

 In order to evaluate the potential differences between the patients included in the two severity groups, a descriptive table was built (Table 1). This table is important to find out whether or not patients’ characteristics could explain the different results found for the two groups regarding the association between immunoglobulin levels and the risk of mortality, as showed later in this section. Table 1 shows that patients were elderly individuals, with the group of patients with SOFA score ≥ 8 having a significant higher proportion of men. The most frequent comorbidities found in both groups of patients were chronic cardiovascular disease, chronic respiratory disease, chronic renal failure and diabetes mellitus. The most severe group of patients showed a significant higher frequency of patients with history of chronic hepatic failure. The proportion of patients with prior immunosuppression did not differ in a significant manner between both groups. Sources of infection were similar in the two groups compared, with predominance of sepsis of respiratory, abdominal and urological origin. Both groups presented also a similar proportion of infections caused by Gram + bacteria and fungi, but the most severe group had a significant higher frequency of infections caused by Gram-negative bacteria along with an overall higher frequency of patients with microbiologically confirmed infection. Ninety-four percentage of the patients with SOFA ≥ 8 presented with cardiovascular dysfunction, compared with 63% in the group of patients with SOFA < 8. As expected, patients in the most severe group showed higher APACHE-II scores, higher creatinine levels in plasma, and lower platelets counts in blood. In addition, mortality at the ICU and also at the hospital was markedly higher in this group. Immunoglobulin and albumin levels in plasma did not differ in a significant manner between both groups of patients. In those patients with positive microbiological identification, proportion of patients receiving appropriated antibiotic treatment based on the antibiogram results was similar between both severity groups (80 vs. 73%, *p* = 0.420).

### Identification of immunoglobulin thresholds associated with mortality

**Fig. 1:**
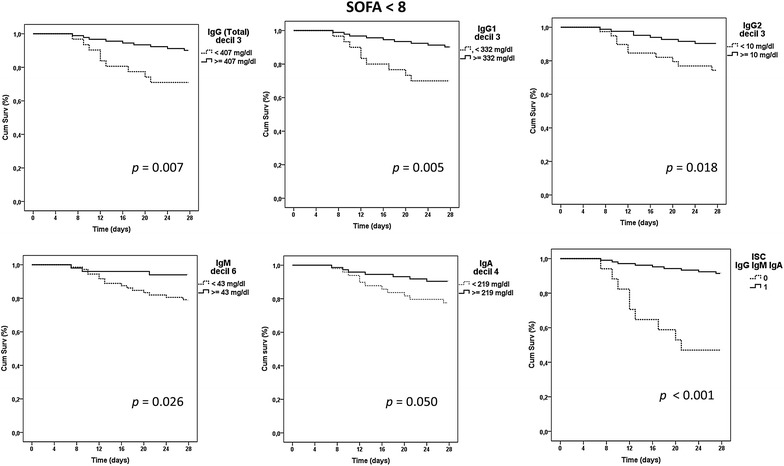
Kaplan–Meier analysis for survival depending on immunoglobulin levels in the group with SOFA < 8

**Fig. 2:**
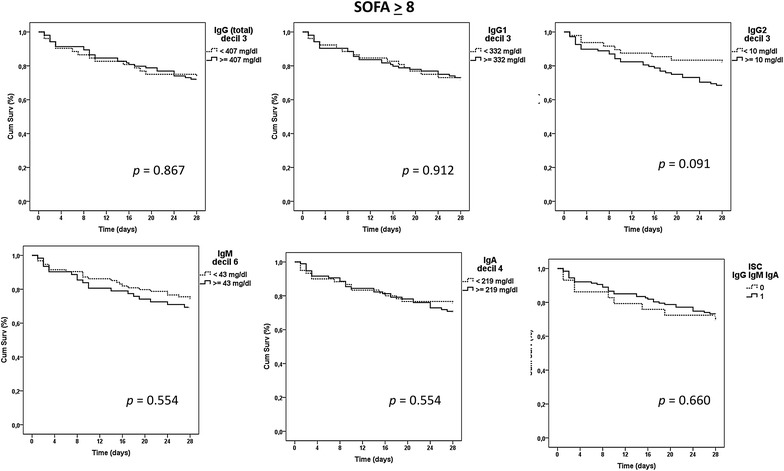
Kaplan–Meier analysis for survival depending on immunoglobulin levels in the group with SOFA ≥ 8

Kaplan–Meier analysis identified five immunoglobulin thresholds associated with mortality in the group of patients with SOFA < 8, corresponding to IgG, IgG1, IgG2, IgM and IgA (Fig. 1). The triple ISC built based upon the thresholds corresponding to the three major immunoglobulin isotypes (IgG, IgM and IgA) showed the highest impact on survival mean time, which was reduced 6.6 days in average in those patients showing a ISC IgGAM = 0 (Fig. 1; Additional file 1). When Kaplan–Meier analysis was repeated for those patients of the most severe group (those with SOFA ≥ 8), no thresholds associated with mortality were identified for individual immunoglobulins (Fig. 2). In addition, the ISC IgGAM failed to show any association with mortality in this analysis (Fig. 2).

### Regression analysis for prediction of mortality risk

To evaluate the impact of the immunoglobulin thresholds on the mortality risk over the acute phase of the disease and also over the post-acute phase of the disease, regression analysis for predicting either ICU or hospital mortality was performed in the group of patients with SOFA < 8:

### ICU mortality

We first compared the proportion of survivors and non-survivors at the ICU in the patients with immunoglobulin levels below/above these thresholds (Table 2):

**Table 2:** Proportion of survivors and non-survivors at the ICU in the group of patients with SOFA score <8 depending on the immunoglobulin thresholds

	Thresholds	SOFA < 8: ICU mortality					
		Patients with prior immunosuppression included (*n* = 122)			Patients with prior immunosuppression excluded (*n* = 99)		
		Non-survivors/total “*n*” in each category	%	*p*	Non-survivors/Total “n” in each category	%	*p*
IgG (total)	<407 mg/dl	8/31	25.8	0.045	6/24	25.0	0.048
	≥407 mg/dl	10/91	14.8		7/75	9.3	
IgG1	<332 mg/dl	8/30	26.7	0.034	6/24	25.0	0.048
	≥332 mg/dl	10/92	14.8		7/75	9.30	
IgG2	<10 mg/dl	9/39	23.1	0.076	8/32	25.0	0.016
	≥10 mg/dl	9/83	10.8		5/67	7.5	
IgM	<43 mg/dl	13/72	18.1	0.217	9/54	16.7	0.254
	≥43 mg/dl	5/50	10.0		4/45	8.9	
IgA	<219 mg/dl	10/49	20.4	0.149	7/39	17.9	0.253
	≥219 mg/dl	8/73	11.0		6/60	10.0	
ISC IgGAM	=0	7/17	41.2	0.001	5/12	41.7	0.002
	=1	11/105	10.5		8/87	9.2	

Those thresholds showing differences at the level *p* < 0.1 in Table 2 are further evaluated for their association with the risk of ICU mortality by using a multivariate analysis (Table 3). This analysis evidenced that exhibiting levels of IgG, IgG1 below their respective thresholds or having an ISC IgGAM = 0 was a robust, independent risk marker of ICU mortality. The highest odds ratio corresponded to the ISC IgGAM. While the presence of patients with prior immunosuppression did not affect the predictive ability of IgG, IgG1 and ISC IgGAM, it did influence IgG2, which was only able to predict mortality in those patients with no previous antecedents of immunosuppression.

**Table 3:** Multivariate logistic regression analysis to evaluate the association between immunoglobulins and the risk of mortality at the ICU in the group of patients with SOFA score <8

Immunoglobulins	SOFA < 8							
	A. Patients with prior immunosuppression included (*n* = 122)				B. Patients with prior immunosuppression excluded (*n* = 99)			
	OR	CI 95%		*p*	OR	CI 95%		*p*
IgG (total) < 407 mg/dl	7.29	1.62	32.79	0.010	9.02	1.45	56.30	0.019
IgG1 < 332 mg/dl	8.02	1.82	35.32	0.006	8.95	1.44	55.79	0.019
IgG2 < 10 mg/dl	3.71	0.93	14.80	0.064	6.22	1.08	35.72	0.040
ISC IgGAM = 0	13.79	2.61	72.98	0.002	17.53	2.23	137.57	0.006

Adjusting variables for *A.* were (diabetes mellitus) (presence of prior immunosuppression) (APACHE-II score) (presence of respiratory infection), (microbiologically confirmed infection). Adjusting variables for *B.* were (APACHE-II score) (presence of respiratory infection), (presence of abdominal infection) (microbiologically confirmed infection)

### Hospital mortality

When hospital mortality was analyzed, only ISC IgGAM showed differences for the proportion of survivors and non-survivors between patients with low and high immunoglobulin levels (Table 4).

**Table 4:** Proportion of survivors and non-survivors at the hospital in the group of patients with SOFA score <8 depending on the immunoglobulin thresholds

	Thresholds	SOFA < 8: Hospital mortality					
		Patients with prior immunosuppression included (*n* = 122)			Patients with prior immunosuppression excluded (*n* = 99)		
		Non-survivors/Total “*n*” in each category	%	*p*	Non-survivors/Total “*n*” in each category	%	*p*
IgG (total)	<407 mg/dl	10/31	32.3	0.119	7/24	29.2	0.209
	≥407 mg/dl	18/91	19.8		13/75	17.3	
IgG1	<332 mg/dl	10/30	33.3	0.119	7/24	29.2	0.209
	≥332 mg/dl	18/92	19.6		13/75	17.3	
IgG2	<10 mg/dl	11/39	28.2	0.344	9/32	28.1	0.175
	≥10 mg/dl	17/83	20.5		11/67	16.4	
IgM	<43 mg/dl	19/72	26.4	0.279	12/54	22.2	0.583
	≥43 mg/dl	9/50	18.0		8/45	17.8	
IgA	<219 mg/dl	14/49	28.6	0.226	10/39	25.6	0.277
	≥219 mg/dl	14/73	19.2		10/60	16.7	
ISC IgGAM	=0	9/17	52.9	0.002	6/12	50.0	0.006
	=1	19/105	18.1		14/87	16.1	

The multivariate analysis confirmed that the ISC IgGAM was a strong predictor of the risk of hospital mortality, not affected by the presence of prior immunosuppression (Table 5).

**Table 5:** Multivariate logistic regression analysis to evaluate the association between immunoglobulins and the risk of mortality at the hospital in the group of patients with SOFA score <8

Immunoglobulins	SOFA < 8							
	A. Patients with prior immunosuppression included (*n* = 122)				B. Patients with prior immunosuppression excluded (*n* = 99)			
	OR	CI 95%		*p*	OR	CI 95%		*p*
ISC IgGAM = 0	7.98	1.94	32.87	0.004	5.63	1.13	28.11	0.035

Adjusting variables for *A.* were (age) (presence of prior immunosuppression) (APACHE-II score) (microbiologically confirmed infection). Adjusting variables for *B* were (APACHE-II score) (respiratory infection) (microbiologically confirmed infection)

Multivariate regression analysis for the ISCs combining two immunoglobulins was not performed since the vast majority of non-survivors showing a “0” in the double ISCs showed also a “0” in the triple ISC (overlap with the triple score was 100% for ISC IgGM, of 90% for ISC IgGA and 82% for ISC IgMA).

### Potential influence of hemodilution on the results

Interestingly, hemodilution had no effect on the results observed in the less severe group of patients, as evidenced by the absence of significant correlation in the Spearman test between levels of immunoglobulins and albumin concentration in plasma (*p* > 0.05).

## Discussion

This study shows for the first time that the influence of endogenous immunoglobulin levels on the prognosis of patients with sepsis seems to be restricted to a subset of individuals presenting at the ICU with limited extent of organ failure. Our results evidenced also that the simultaneous presence of low levels of IgG, IgA and IgM was found to be a consistent predictor of both acute mortality (at the ICU) and post-acute mortality (at the hospital) in these patients, independently of the presence or absence of previous immunosuppression. In contrast, the ability of total IgG or IgG1 to predict mortality was restricted to the acute period of the disease (ICU mortality). While the presence of patients with previous immunosuppression did not alter the predictive ability of total IgG and IgG1, it modified that of IgG2, which was only able to predict ICU mortality in those patients with no previous immunosuppression. These findings reinforce the superiority of the combined immunoglobulin score over immunoglobulins individually considered to identify sepsis patients at risk of poor outcomes [4].

In the era of precision medicine in sepsis [11, 12], defining the immunological state of the patient will be crucial to the success of any biological response modifier for sepsis [13, 14]. Our findings could contribute to personalize treatment with IVIG in this disease. Failure of IVIG in demonstrating clinical benefit in sepsis [15] could be explained by different factors concerning both the patient and the IVIG preparation, which have already been discussed elsewhere [1, 16]. Our results provide new clues to better design future trials with IVIG in sepsis and/or to improve data analysis from these trials. The more relevant would be that sepsis patients should not be considered as a homogenous population. The impact of IVIG on the outcome of the patients should be analyzed stratifying the patients by their levels of endogenous immunoglobulins and by the degree of disease severity at ICU admission. Quantification of immunoglobulin levels using nephelometry is a fast test which takes less than 2 h. In turn, the SOFA score has widespread familiarity within the critical care community, making it useful to assess the degree of organ failure extent at ICU admission [7, 17]. In addition, our results provide a scientific rational to evaluate whether IVIG preparations containing IgG, IgA and IgM could be more effective for the treatment of sepsis than those containing exclusively IgG [18–20]. Interestingly, Kreymann KG et al., a meta-analysis in 2007 of all randomized controlled studies published on polyvalent immunoglobulins for treatment of sepsis or septic shock, observed a strong protective trend in favor of an immunoglobulin preparation containing the three major immunoglobulin isotypes [21].

As a major limitation of our study, immunoglobulins were measured in samples already available from the GRECIA and the ABISS studies, and in consequence, it was retrospective in nature. This makes that sample size was not calculated based on a predefined primary outcome, which may represent a source of bias. Another limitation was the absence of lactate registries in many patients, which precluded identifying those patients fulfilling the new definition of septic shock as proposed by the SEPSIS-3 consensus [8]. In consequence, new prospective studies should confirm the results obtained in this work.

## Conclusions

Results from this study suggest that endogenous immunoglobulin levels may have a different impact on the mortality risk of sepsis patients based on their severity. Future studies should be directed to investigate whether IVIG therapy may particularly benefit subsets of patients with moderate organ failure extent and low levels of the three immunoglobulin isotypes.

## Additional file

**Additional file 1.** Mean survival time (days) in the SOFA <8 group based on immunoglobulin thresholds. Differences between groups were assessed using the log-rank test. Δ (days) represents [(mean survival time in patients with levels of immunoglobulin above the threshold) – (mean survival time in patients with levels of immunoglobulin below the threshold)]. Time was censored at 28 days following ICU admission

## Abbreviations

ABISS-Edusepsis: AntiBiotic Intervention in Severe Sepsis
APACHE-II: Acute Physiology and Chronic Health Evaluation II Score
GRECIA: Grupo de Estudios y Análisis en Cuidados Intensivos
IVIG: intravenous immunoglobulin
ISC IgGAM: immunoscore combining IgG, IgA, IgM
ISC IgGM: immunoscore combining IgG, IgM
ISC IgGA: immunoscore combining IgG, IgA
ISC IgMA: immunoscore combining IgM, IgA
SBITs: Score-based immunoglobulin G therapy of patients with sepsis
SOFA: Sequential Organ Failure Assessment Score
