# Supplementary material for: The protective association of endogenous immunoglobulins against sepsis mortality is restricted to patients with moderate organ failure
Source: Ann Intensive Care. 2017 Apr 20;7:44. doi: 10.1186/s13613-017-0268-3 (PMC5399013; doi:10.1186/s13613-017-0268-3)
Supplement: Supplementary file 1 — Additional file 1. Mean survival time (days) in the SOFA <8 group based on immunoglobulin thresholds. Differences between groups were assessed using the log-rank test. Δ (days) represents [(mean survival time in patients with levels of immunoglobulin above the threshold) – (mean survival time in patients with levels of immunoglobulin below the threshold)]. Time was censored at 28 days following ICU admission [file 13613_2017_268_MOESM1_ESM.doc]

|  | ***SOFA < 8*** | | | | | |
| --- | --- | --- | --- | --- | --- | --- |
|  | **< threshold** | | **> threshold** | | **∆**  **(days)** | ***p*** |
|  | **n** | **days [CI95%]** | **n** | **days [CI95%]** |  |  |
| **IgG (total) = 407 mg/dl** | 31 | 23.7 [21.2 – 26.2] | 91 | 26.8 [25.9 – 27.6] | 3.1 | **0.007** |
| **IgG1 = 332 mg/dl** | 30 | 23.6 [21.1 – 26.2] | 92 | 26.8 [26.0 – 27.7] | 3.2 | **0.005** |
| **IgG2 = 10 mg/dl** | 39 | 24.5 [22.4 – 26.6] | 83 | 26.7 [25.8 – 27.6] | 2.2 | **0.018** |
| **IgM = 43 mg/dl** | 72 | 25.3 [24.0 – 26.7] | 50 | 27.1 [25.9 – 28.2] | 1.8 | **0.026** |
| **IgA = 219 mg/dl** | 49 | 25.1 [23.53 – 26.8] | 73 | 26.7 [25.7 – 27.7] | 1.6 | **0.050** |
| **ISC IgGAM (0/1)** | 17 | 20.3 [16.5 – 24.1] | 105 | 26.9 [26.2 – 27.7] | 6.6 | **0.001** |
